# Zip4 Mediated Zinc Influx Stimulates Insulin Secretion in Pancreatic Beta Cells

**DOI:** 10.1371/journal.pone.0119136

**Published:** 2015-03-25

**Authors:** Alexandre B. Hardy, Kacey J. Prentice, Sean Froese, Ying Liu, Glen K. Andrews, Michael B. Wheeler

**Affiliations:** 1 Department of Physiology, University of Toronto, Toronto, Ontario, Canada; 2 University of Kansas Medical Center, Kansas City, Kansas, United States of America; University of British Columbia, CANADA

## Abstract

Zinc has an important role in normal pancreatic beta cell physiology as it regulates gene transcription, insulin crystallization and secretion, and cell survival. Nevertheless, little is known about how zinc is transported through the plasma membrane of beta cells and which of the class of zinc influx transporters (Zip) is involved. Zip4 was previously shown to be expressed in human and mouse beta cells; however, its function there is still unknown. Therefore, the aim of this study was to define the zinc transport role of Zip4 in beta cells. To investigate this, Zip4 was over-expressed in MIN6 beta cells using a pCMV6-Zip4GFP plasmid. Organelle staining combined with confocal microscopy showed that Zip4 exhibits a widespread localization in MIN6 cells. Time-lapse zinc imaging experiments showed that Zip4 increases cytoplasmic zinc levels. This resulted in increased granular zinc content and glucose-stimulated insulin secretion. Interestingly, it is unlikely that the increased glucose stimulated insulin secretion was triggered by a modulation of mitochondrial function, as mitochondrial membrane potential remained unchanged. To define the role of Zip4 *in-vivo*, we generated a beta cell-specific knockout mouse model (Zip4BKO). Deletion of the Zip4 gene was confirmed in Zip4BKO islets by PCR, RT-PCR, and immuno-histochemistry. Zip4BKO mice showed slightly improved glucose homeostasis but no change in insulin secretion during an oral glucose tolerance test. While Zip4 was not found to be essential for proper glucose homeostasis and insulin secretion *in vivo* in mice, this study also found that Zip4 mediates increases in cytoplasmic and granular zinc pools and stimulates glucose dependant insulin secretion *in-vitro*.

## Introduction

The highest cellular zinc concentration in the body is within pancreatic beta cells where it plays an essential role in insulin processing, including biosynthesis, secretory granule maturation, and exocytosis [1, 2]. The essential role of zinc in regulating human beta cell function was first identified based on genome-wide association studies that identified zinc efflux transporter 8 (ZnT8) as a significant risk factor for type 2 diabetes [3–7]. ZnT8 is localized on insulin secretory vesicles and is the primary transporter that moves zinc from the cytoplasm into these organelles, explaining its requirement for insulin crystallization and proper insulin secretion [8–12]. While ZnT8, and thus zinc entry into insulin secretory granules, has been extensively characterized, our knowledge on how zinc enters beta cells through the plasma membrane, and the zinc influx transporters (Zip) involved is still limited. To date, all that is known is that some zinc enters beta cells through voltage gated calcium channels [13].

The Slc39a1-Slc39a14 genes encode for the Zip family. Zips are localized either in the cell plasma membrane or in organelle membranes. In the plasma membrane, Zip function is to transport extracellular zinc into the cytosol. In organelles, Zips transport zinc from intra-organelle stores to the cytosol [14, 15]. In beta cells, little is known about the functions of Zips. It was found that mouse beta cells express Zip6, 7, and 8, which are dynamically regulated by extracellular glucose [16]. Mouse islets also display some expression of Zip1, Zip9, and Zip14 [13, 17]. Interestingly, quantitative real-time polymerase chain reaction (qPCR) detected mRNA expression of Zip4 in human islets [18] and immuno-histochemistry performed in one study reported that Zip4 is also expressed in mouse islet beta cells [19]. In enterocytes, a Zip4 loss of function mutation is linked to a disruption of zinc transport through the apical membrane, which leads to a loss of zinc absorption by the intestine. In mice, an enterocyte-specific knockout of the Zip4 gene mimics a disorder similar to *acrodermatitis enteropathica* confirming the pivotal role of Zip4 in the regulation of zinc absorption [20].

However, Zip4 function in islet beta cells is unknown. Therefore, in the current study, we aimed to confirm Zip4’s role as a zinc transporter that transports zinc through the plasma membrane to enter the cytosol in beta cells.

We first studied Zip4's role *in vitro* in beta cells by overexpressing Zip4 in the mouse insulinoma beta cell line MIN6. Zip4 exhibits a diffuse pattern in the whole cell without specific localization in the plasma membrane, mitochondria or endoplasmic reticulum. Zinc imaging experiments were performed to define the role of Zip4 in zinc uptake. Imaging showed that Zip4 promotes an increased accumulation of cytoplasmic zinc, which was correlated to augmented granular zinc content. This increase in the cytoplasmic zinc pool did not change insulin biosynthesis or total insulin content. Nevertheless, insulin secretion was elevated when Zip4 was over-expressed. To further study the origin of this increased insulin secretion, mitochondrial membrane potential (MMP) was monitored and revealed an unchanged glucose-induced hyperpolarization of the MMP. This suggests that the increased insulin secretion is not linked to a modulation of mitochondrial fuel-mediated insulin secretion. Since Zip4 up-regulation *in vitro* modulated intracellular zinc pools and increased insulin secretion, we wanted to know the function of Zip4 *in vivo*. We confirmed Zip4 specific localization in islets by performing immuno-cytochemistry on mouse pancreatic sections. We then generated a new beta cell-specific Zip4 knockout mouse model (Zip4BKO). We confirmed the deletion of the Zip4 gene and showed that Zip4 deletion in mouse beta cells is associated with a slight improvement of glucose homeostasis without changes in insulin secretion. Profiling of other zinc transporters in Zip4BKO islets evidenced an increased expression of Znt8 mRNA. This Znt8 expression change could represent a compensation mechanism that might have masked a phenotype in Zip4 in knockout mice.

## Materials and Methods

### Animal care and generation of Zip4 knockout mice

**Fig 1:**
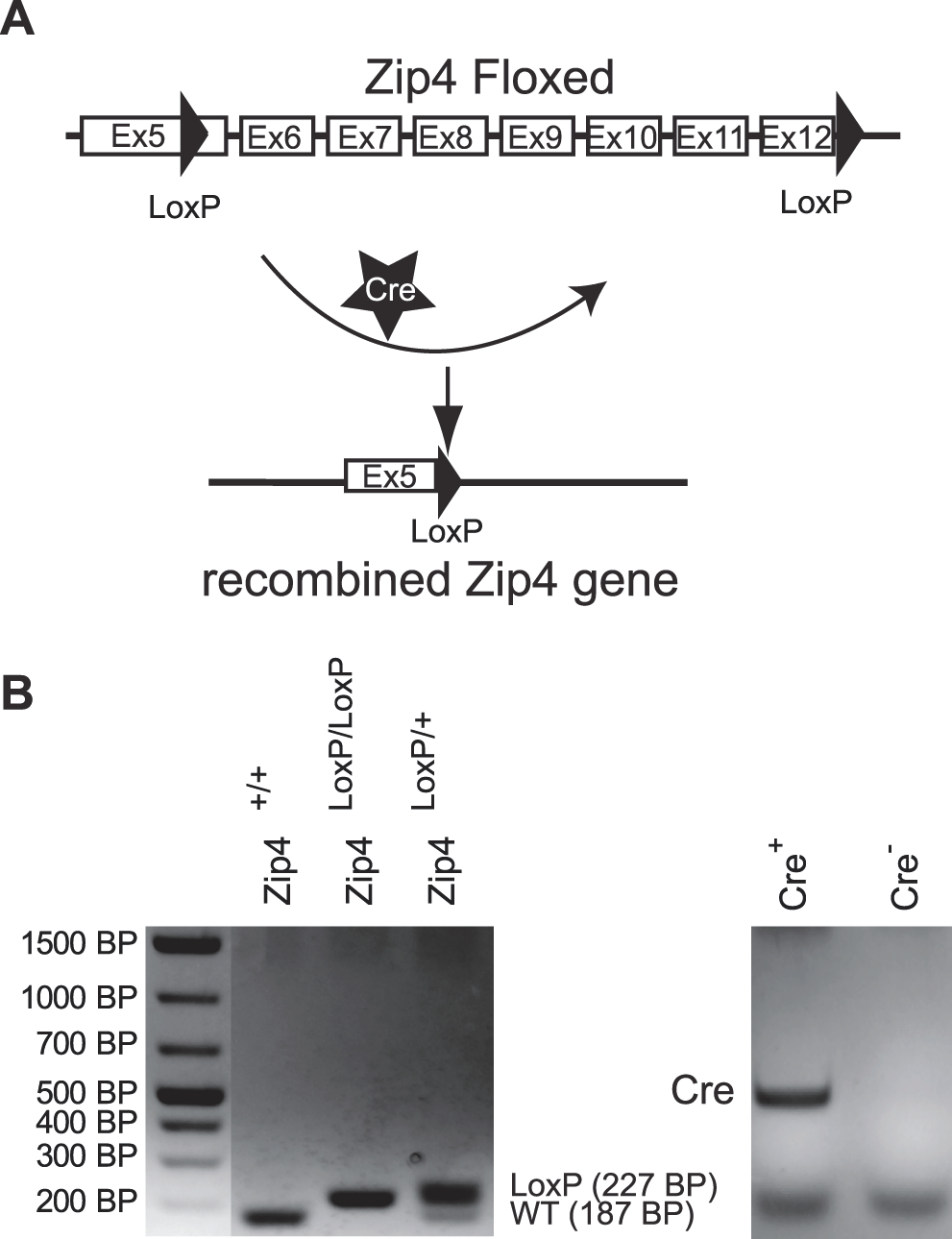
Generation of Zip4BKO mice. A. Schematic of the targeting construct of Zip4 showing the region of the Zip4 gene flanked by the loxP sites. The cre recombinase induced recombination at these loxP sites which results in the excision of the portion of the Zip4 gene that contains 7 exons. B. PCR results obtained from ear skin samples.

The Animal Care Committee at the University of Toronto approved all experiments. Animals were handled according to the guidelines of the Canadian Council of Animal Care. Deletion of the Zip4 gene was achieved using the Cre/lox recombination system. Briefly, LoxP sites were inserted in exon 5 and downstream of exon 12 of the Zip4 gene (Fig. 1A). Cre activity is controlled by the pancreatic beta cell specific Ins2 promoter. Therefore, deletion of the portion of Zip4 gene from exon 5 through exon 12 will occur specifically in beta cells when these mice are crossed (Fig. 1B).

Our beta cell-specific Zip4 mouse model was generated by crossing TgN(Ins2-Cre)25Mgn mice (maintained on an hybrid C57BL/6/129J background) to Zip4loxP/loxP mice (hybrid C57BL/6J/29sv backcrossed six times onto C57BL/6J mice). Then, Ins2Cre+/+ and Zip4+/loxP were interbred for three generations. Offspring expressing the Cre transgene alone were used as control mice (RipCre). From the same litters, offspring expressing the Cre transgene and Zip4loxP/loxP were used as beta cell-specific Zip4KO mice and termed Zip4BKO. Therefore, control RipCre mice and Zip4BKO mice were littermates and had the same mixed genetic background. 6 to 8-week-old male mice were used for all experiments. Mice were genotyped using skin DNA and standard multiplex PCR using flox and Cre primers (Table 1).

**Table 1:** PCR primer sequences used for mouse genotyping and qPCR.

**Gene**	**Forward**	**Reverse**	**Accession Number**
*β-actin*	*CTGAATGGCCCAGGTCTGA*	*CCCTGGCTGCCTCAACAC*	NM_007393
*Cre*	*GGCAGTAAAAACTATCCAGCAA*	*GTTATAAGCAATCCCCAGAAATG*	AB542060
*Flox*	*AGGAGGAAGAGTAGTGGATTTCAAGG*	*CGAGCCATAGAGATACCCTGTGG*	NM_028064.2
*Flox (post Cre)*	*AGGAGGAAGAGTAGTGGATTTCAAGG*	*CTTTTCTGGATTCATCGACTGTGG*	
*Kcnj11*	*GACATCCCCATGGAGAATGG*	*TCGATGACGTGGTAGATGATGAG*	NM_010602
*Pc1*	*AATGGGCGGCGGAGAT*	*CCAAAAGGTCATACCCCAGTTC*	NM_013628
*Pc2*	*GCTGGACCAGCCCTTTATGA*	*ATGTCGATCAGTTGAGGCATGT*	NM_008792
*Cpe*	*CTGATCCACAGCACCCG*	*AAT GCGATGCGGCTTTCTCA*	NM_013494
*Ins1*	*TGGCTTCTTCTACACACCCAAGTC*	*ACTGATCCACAATGCCACGCTTCT*	NM_008386
*Zip1*	*ACCGAGTTATCACAGCCACC*	*TGGTTAGCACCTGACCTTCG*	NM_013901.2
*Zip2*	*AGGAGTAAAAATCGGCTGCCT*	*TGTAGCTGCATCCATCTGGAAC*	NM_001039676.2
*Zip3*	*TGTAGCCAGGAATACGCCAC*	*TGCCTGTGAAGGTCATCGAG*	NM_134135.1
*Zip4*	*CGGGCCACCAGCGTATTTA*	*AGGGACTTGTGTTGGCTCTG*	NM_028064.2
*Zip5*	*ATGGTCAGCCAACGAATGGT*	*TGGACATCTGGGCAGGAATG*	NM_001136237.1
*Zip6*	*TGGGCGAGATCCTTTCCCTA*	*CAGTCACACGGTTGCTGGTA*	NM_139143.3
*Zip7*	*GACAGTGTCCAGGTGGTGTT*	*CATGGCCACTCCCACGATAG*	NM_008202.2
*Zip8*	*AACCAGCTCGAACTTCTCTGC*	*GAAAGACTGGGCTTTGCGTTG*	NM_026228.5
*Zip9*	*GCAGTTCCAAAATCACCACCA*	*TTGCCCAAGAGCATTCAGTGT*	NM_026244.2
*Zip10*	*TCACTGTGAGCAACGGAGTC*	*GCTGAACTGCCCATCAAAGC*	NM_172653.2
*Zip11*	*CTGCCCGCTGGAGAATATGA*	*CAAGGTTACAGCTCCGTGGT*	NM_001166503.1
*Zip12*	*TCCTCTACTGAGACCGGCAA*	*CAGTGGTCACCAGCAGAGAG*	NM_001012305.2
*Zip13*	*TGACTGTAACAGGGTCCCCA*	*CTGCCTGTCGCCTGGATAAT*	NM_026721.
*Zip14*	*TCTGGTTTTCTGAGGTGCAGG*	*GCTCTGGAGACCTCTTTGCG*	NM_001135152.1
*Znt8*	*AAGCCTGACTACAAAATTGCTGATC*	*GACGGTGCTGGCCAAAAC*	NM_172816.3

### Islet isolation and dispersion

Mouse islets were isolated by collagenase digestion of the mouse pancreas and dispersed as described [21].

### qPCR

qPCR analysis was performed as previously described [22]. Primers were designed using Primer Express version 2.0 software (Applied Biosystems) and are listed in Table 1. Data were normalized to mouse β-actin mRNA.

### Oral glucose tolerance test (OGTT)

Following a 6-h fast, glucose (1.5 g/kg body weight) was given by oral gavage, and blood glucose was measured at 0, 10, 20, 30, 60, and 120 min from tail vein blood with a glucometer. Blood was centrifuged at 8,000 rpm for 10 min at 4°C. Supernatant (plasma) was used to measure insulin by ELISA (ALPCO). Area under the curve was calculated using GraphPad Prism software.

### Cell culture

MIN6 cells [23] were cultured in 25mM glucose Dulbecco’s modified Eagle’s medium (500 mL supplemented with 10% fetal bovine serum, 100 units/ml penicillin/G sodium, 100 μg/ml streptomycin sulphate, 1.7 μL β-mercaptoethanol and without sodium pyruvate).

### Slc39a4 overexpression in MIN6 cells

The Slc39a4 (Zip4) gene was over-expressed using a pCMV6 plasmid carrying Slc39a4 fused to turbo GFP (pCMV6-Zip4GFP; Origene; Slc39a4 NM_028064 Mouse cDNA ORF Clone). MIN6 cells used for cytoplasmic zinc imaging were seeded on 22mm coverslips in 6 well culture plates. After 48h culture, 400 μL of opti-MEM reduced serum media containing 2 μL of Lipofectamine 2000 and 0.5 μg of pCMV6-Zip4GFP was added to 1 mL of Dulbecco’s modified Eagle’s medium supplemented with 10% fetal bovine serum with 50 units/ml penicillin/G sodium, 50 μg/ml streptomycin sulphate. MIN6 cells used for granular zinc and MMP imaging were also seeded on 22mm coverslips in 6 well culture plates and cultured following the protocol detailed above, and Zip4 was overexpressed with 5 μL of Lipofectamine 2000 and 2.5 μg of pCMV6-Zip4GFP. MIN6 control cells were transfected with 2.5 μg of control pcDNA-eGFP plasmid and 5 μL of Lipofectamine 2000. For glucose-stimulated insulin secretion (GSIS), MIN6 cells were seeded in 96 well plates and cultured for 24h. Then, 10 μL of opti-MEM reduced serum media containing 0.1 μL of Lipofectamine 2000 and 0.034 μg of pCMV6-Zip4GFP was added to 50 μL of Dulbecco’s modified Eagle’s medium supplemented with 10% fetal bovine serum with 50 units/ml penicillin/G sodium, 50 μg/ml streptomycin sulphate. After 72h transfection, the media was replaced with fresh Dulbecco’s modified Eagle’s medium supplemented with 10% fetal bovine serum, 50 units/ml penicillin/G sodium, 50 μg/ml streptomycin sulphate and MIN6 cells were allowed to recover by culturing them for an additional 12h. Control MIN6 cells used for GSIS were transfected with 0.1 μL of Lipofectamine 2000 and 0.034 μg of pcDNA-eGFP following the protocol detailed above.

### Immuno-histochemistry, dithizone and cellular compartment staining

Pancreatic section were obtained, processed and stained with an anti-mouse polyclonal Zip4 antibody (1/200; Pierce antibody products, ThermoScientific) as previously described [10]. Dispersed islet cells were fixed in 4% (vol./vol.) paraformaldehyde for 15 min, permeabilized with 0.2% (vol./vol.) Triton-X-100 for 10 min and blocked overnight in 5% (wt/vol.) BSA and 0.1% (vol./vol.) Triton-X-100 at 4°C. Cells were then labelled overnight at 4°C with a rabbit polyclonal anti-Zip4 (1:1000) antibody described in [24], anti-insulin (0.05 mol/l; DAKO, Glostrup, Denmark), and monoclonal anti-glucagon (1:300; Sigma) antibodies. Cells were then stained at room temperature for 30 minutes with Cy5-conjugated anti-rabbit, FITC-conjugated anti-guinea pig or FITC-conjugated anti-mouse secondary antibodies (Jackson ImmunoResearch Laboratories, West Grove, PA, USA) respectively. Next, cells were washed and mounted on glass slides. Images were acquired using a software package (LSM510; Zeiss, Thornwood, NY, USA) on a confocal microscope with 488/633 nm excitation laser line and 545 nm beam-splitter and a 63X objective. Dithizone staining was performed by incubating islets in a solution of PBS-dithizone solution at room temperature for 20 minutes. Color images of islets were taken on a Zeiss Axioplan 2 imaging system. MIN6 cell plasma membrane was stained with 30 μg/mL of WGA Alexa Fluor 555 conjugate (Invitrogen) for 5 min at room temperature in DPBS. MIN6 cell mitochondria were stained with 0.5 μM MitoTracker Red CMXRos (Invitrogen) in a cell culture incubator for 45 min. Then, cells were rinsed three times in DPBS and fixed in a solution of 4% PFA for 15 min. Once fixed, cells were permeabilized with 0.2% (vol./vol.) Triton-X-100 for 15 min. Nuclei were revealed by incubating MIN6 cells with TO-PRO-3 (2 μg/mL) for 15 min at room temperature. To stain the endoplasmic reticulum, MIN6 cells transfected with pCMV6-Zip4GFP were blocked overnight in 5% (wt/vol.) BSA and 0.1% (vol./vol.) Triton-X-100 at 4°C. The endoplasmic reticulum was stained using a primary rabbit KDEL antibody (1/200 dilution in PBS-1%BSA-0.1%Triton-X-100 (vol./vol.)) incubated with MIN6 cells for 12h at 4°C. Then, a donkey Alexa 555 anti-rabbit antibody ((1/500 dilution) in PBS-1% BSA-0.1% Triton-X-100 (vol./vol.) was applied to the cells for 2h at room temperature. Once stained, MIN6 cells were mounted with the anti-fade pro-Gold mounting media (Invitrogen) and a coverslip. Images were acquired using a software package (LSM510; Zeiss, Thornwood, NY, USA) on a confocal microscope with 488/633 nm excitation laser line and 545 nm beam-splitter and a 63X objective. Background staining was removed from the mitotracker red images. Then co-localization was calculated with the Pearson’s correlation coefficient using the Image J software package [25].

### Glucose stimulated insulin secretion (GSIS)

Briefly, MIN6 cells were preincubated for 60 minutes in a glucose-free Krebs-Ringer HEPES buffer (125 mM NaCl, 5.9 mM KCl, 1.28 mM CaCl2, 5.0 mM NaCO3, 25 mM HEPES, and 0.1% (w/v) bovine serum albumin, KRB). Following this preincubation, MIN6 cells were incubated for 90 min with KRB without glucose, or with KRB containing 10 mM D-glucose. Insulin from the supernatant was measured using a fluorescence resonance energy transfer based immunofluorescent assay following the manufacturer’s protocol (Cisbio US). Total insulin content was measured by applying two cycles of freeze-thaw in a −80 freezer to lyse the MIN6 cells. Then, insulin was measured using the same fluorescence resonance energy transfer based immunofluorescent assay.

### Zinc imaging

Fluorescent imaging experiments were performed in imaging buffer (130 mM NaCl, 5 mM KCl, 2 mM CaCl2, 1 MgCl2, 5 mM NaHCO3, 10 mM Hepes) supplemented with 200 nM of Tetrakis-(2-Pyridylmethyl) ethylenediamine (TPEN). Fluozin-3AM was used to measure zinc levels in the cytoplasm of MIN6 cells. MIN6 cells were incubated with 2 μM Fluozin-3AM and incubated 45 min in a cell culture incubator. Fluozin-3AM was excited with a 480 nm wavelength and emission was measured with 525 nm band pass filter using 505 nm beam splitter. Following zinc supplementation, the rate of zinc production was calculated by applying a linear curve fit and calculating the slope over the first 400 sec of the fluorescence increase with the Igor software (Wavemetrics), as was the area under the curve. Granular zinc content was estimated with the low affinity zinc dye zinquin. MIN6 cells were incubated with 2 μM zinquin in imaging media in the absence of glucose. Zinquin emission was monitored using 365-nm excitation, 375-nm beam splitter, and 385-nm long-pass filter.

### Mitochondrial membrane potential

Imaging experiments were performed with a similar imaging buffer solution described in the zinc imaging methods section above. MIN6 cells were washed three times with imaging buffer and incubated with 10 μg/mL of rhodamine123 for 15 min in the absence of glucose. After rhodamine123 incubation, MIN6 cells were rinse 3 times with 2 mL of imaging buffer. The experiment was performed on a microscope set-up described previously [13]. An excitation wavelength of 545 nm was used combined with a 550 nm dichroic filter and 600–650 nm band pass emission filter [26].

### Statistical analysis

Data are expressed as means ± SE. Significance was determined using Student’s t-test or two-way repeated measures ANOVA with Tukey-Kramer or Bonferroni’s post-hoc test for OGTT results. cc ˂0.05 was considered statistically significant.

## Results

### Role of Zip4 in MIN6 beta cells

**Fig 2:**
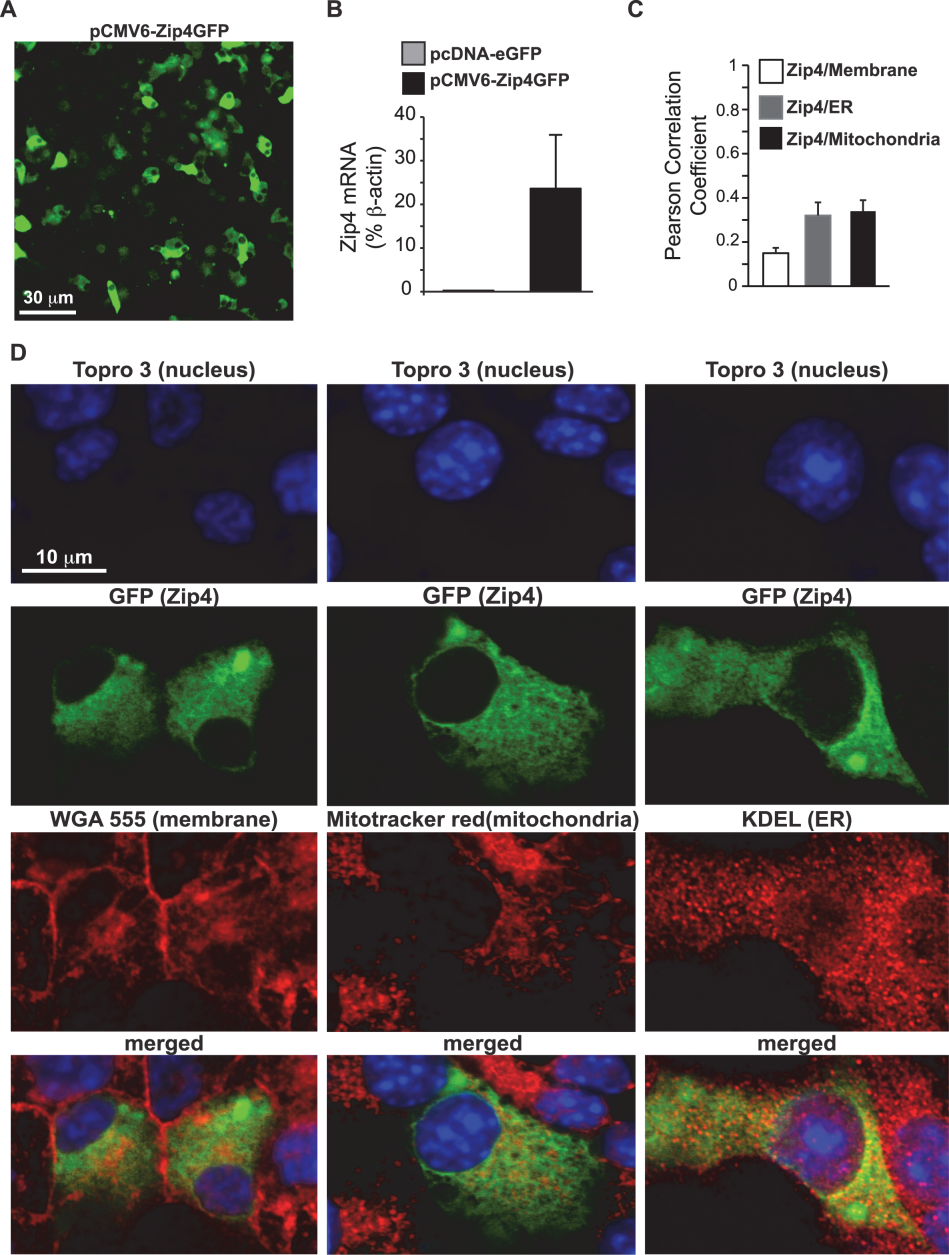
Localization of Zip4 in MIN6 cells. A. Field of view of MIN6 cells transfected with the pCMV6-Zip4GFP plasmid. B. Expression of Zip4 was measured by qPCR performed on MIN6 cells transfected with pCMV6-Zip4GFP plasmid C. Co-localization was estimated with the Pearson correlation coefficient analysis calculated from confocal images obtained from the FITC and Cy3 channel. D. Confocal images of pCMV6-Zip4GFP transfected MIN6 cells after staining of the nucleus with TOPRO-3, plasma membranes with WGA Alexa Fluor 555, mitochondria with MitoTracker Red CMXRos or the endoplasmic reticulum (ER) with immunostaining of KDEL.

MIN6 cells were transfected with a pCMV6-Zip4GFP plasmid. As GFP is fused to the c-terminal end of Zip4, it can be localized with a fluorescent capable microscope (Fig. 2A). qPCR was performed on control and Zip4 plasmid transfected MIN6 cells. qPCR demonstrated that transfection significantly increased expression of Zip4 (Fig. 2B; 3 independent experiments). Confocal microscopy experiments combined with organelle staining were performed to precisely locate Zip4 in beta cells. Subcellular localization of Zip4 was studied using co-staining for plasma membrane, mitochondria and endoplasmic reticulum (ER). Pearson’s correlation calculations were performed to evaluate the level of co-localization between Zip4 and various organelles. Plasma membrane was stained with the WGA Alexa Fluor 555 conjugate stain (Invitrogen). Interestingly, Zip4 was not specifically localized on the membrane of beta cells (Fig. 2D; left panels) as an average Pearson Correlation Coefficient (PCC) of 0.149±0.024 was calculated, (Fig. 2C; n = 12, 3 independent experiments). Zip4 had a diffuse staining pattern and was not specifically localized in any organelle investigated as confirmed by low overall PCC values (Fig. 2C, Fig. 2D; middle panels). Mitotracker Red CMXRos dye was used to stain mitochondria and revealed a low PCC value of 0.339±0.045 between Zip4 and stained mitochondria (Fig. 2C; n = 12, 3 independent experiments). ER was revealed by immunostaining for the ER retention c-terminal sequence KDEL (Fig. 2D; right panels). Confocal images and an average PCC value of 0.32±0.011 suggest that Zip4 is also not specifically localized on the ER (Fig. 2C; n = 6, 3 independent experiments).

**Fig 3:**
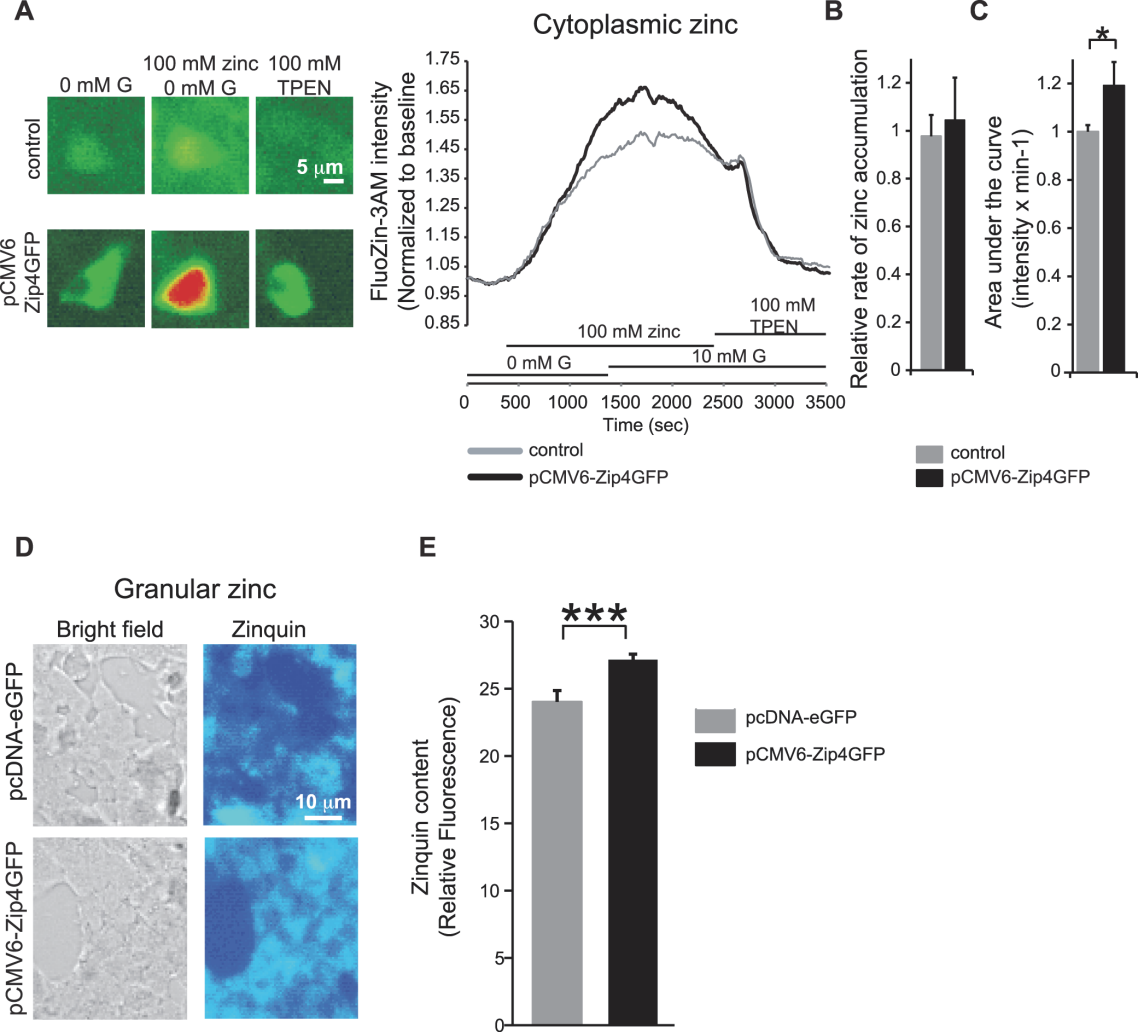
Role of Zip4 in zinc transport in MIN6 cells. A. Fluorescent images (left panels) and 2 representative traces (right panel) of zinc cytoplasmic levels measured with FluoZin-3AM in MIN6 control and pCMV6-Zip4GFP transfected MIN6 cells. B. Following supplementation of 100 μM Zinc, the rate of zinc increase was calculated by applying a linear curve fit and calculating its slope over the first 400 seconds of the fluorescence increase. C. The overall area under the FluoZin-3AM curve in response to 100 μM Zn^2+^ was calculated in control MIN6 and pCMV6-Zip4GFP transfected MIN6 cells. D. Granular zinc content was estimated with the low affinity zinc dye zinquin in MIN6 cells transfected with pcDNA-eGFP (control condition) and pCMV6-Zip4GFP. E. Overall zinquin intensity in control MIN6 and pCMV6-Zip4GFP transfected MIN6 cells. *. p˂0.05; ***. p˂0.001.

Zinc fluorescent imaging experiments were performed to study the role of Zip4 in the regulation of zinc pools in MIN6 beta cells. Fluozin-3AM is a fluorescent zinc probe that detects the cytoplasmic zinc pool. Upon addition of zinc, we found that overall zinc levels were increased by 18% when Zip4 was overexpressed (Fig. 3A-C; n = 34, 2 independent experiments). As zinc is known to specifically accumulate in insulin granules, we estimated the total granular zinc content with the zinc dye zinquin. Interestingly, MIN6 cells overexpressing Zip4 also displayed a significant increase of granular zinc content (Fig. 3D, E, n = 34–44, 3 independent experiments).

**Fig 4:**
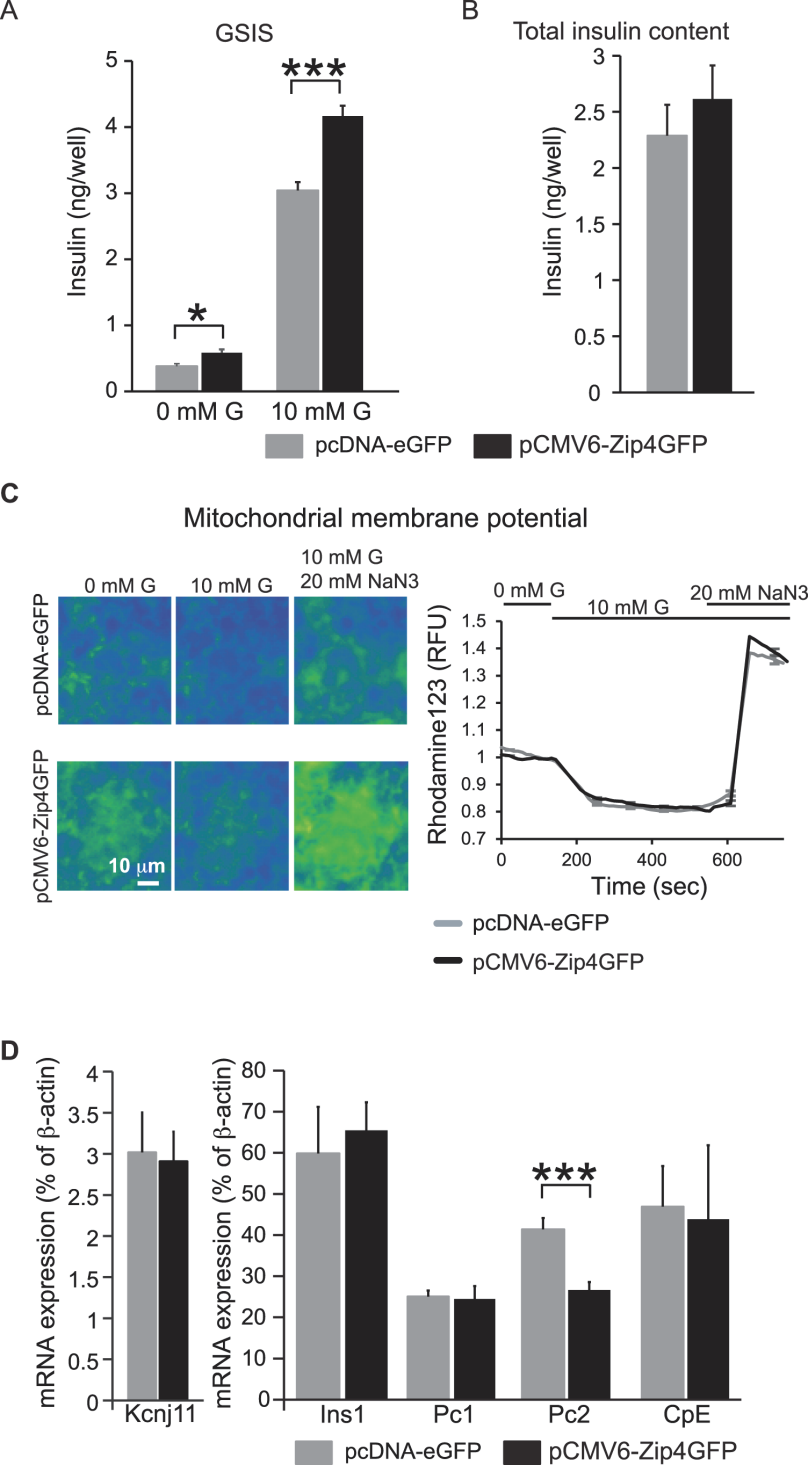
Role of Zip4 in insulin secretion in MIN6 cells. A. Glucose stimulated insulin secretion (GSIS) from MIN6 transfected with control vector pCMV-eGFP and MIN6 cells overexpressing Zip4 (pCMV6-Zip4GFP) in presence (10 mM G) or absence of glucose (0 mM G). B. Total insulin content. C. Representative images (left panels) and average mitochondrial membrane potential traces (right panel) monitored in MIN6 cells with Rhodamine123 in absence of glucose (0 mM G), 10 mM glucose (10 mM G), and 10 mM glucose and 20 mM sodium azide (NaN3). D. mRNA expression of genes indicated in MIN6 cells.*. p˂0.05; ***. p˂0.001.

We studied the role of Zip4 in GSIS and insulin biosynthesis. The insulin secretion in control MIN6 cells in presence of 10 mM glucose (high glucose) was 3.04±0.09 ng/well. Zip4 overexpression in MIN6 cells significantly increased insulin secretion to 4.16±0.16 ng/well (Fig. 4A, 12–16 replicates per condition, 3 independent experiments). Despite this increase, total insulin content was unchanged when Zip4 was overexpressed (Fig. 4B, 12–16 replicates per condition, 3 independent experiments). Mitochondrial respiration was estimated by measuring mitochondrial membrane potential (MMP). Overexpression of Zip4 did not change glucose-induced hyperpolarization of MMP or the overall mitochondrial capacity, tested by adding 20 mM NaN_3_ (Fig. 4C, n = 200, 3 independent experiments). Overexpression of Zip4 also did not change mRNA expression of insulin (Ins1), or the insulin processing enzymes prohormone convertase (Pc) 1 and endoprotease carboxypeptidase E enzyme (CpE) (Fig. 4D, 3 independent experiments). Interestingly, Pc2 mRNA expression was decreased (Fig. 4D, 3 independent experiments). Because we observed that up-regulation of Zip4 in MIN6 cells produced an increase in cytosolic zinc accumulation along with a stimulation of insulin secretion in the presence of high glucose, we then studied the function of Zip4 *in vivo*.

### Zip4 localization in the pancreatic tissue

**Fig 5:**
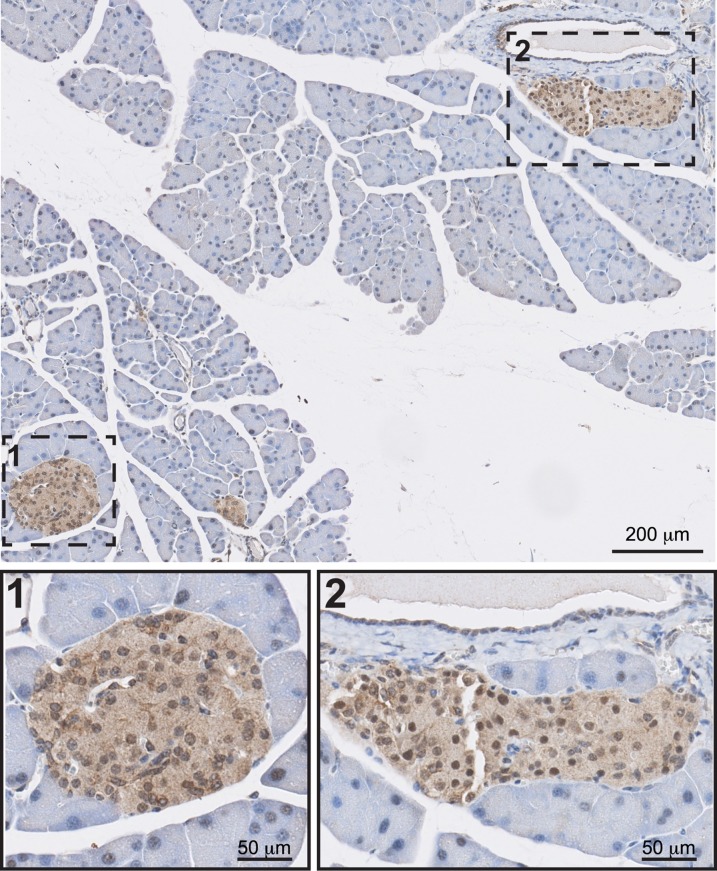
Localization of Zip4 in the pancreas. Bright field images of Zip4 immuno-cytochemistry in mouse pancreatic sections.

To locate Zip4 specifically in the pancreatic tissue, mouse pancreatic sections were stained with a polyclonal Zip4 antibody. Immuno-cytochemistry revealed Zip4 protein expression specifically in islets and its absence from the acinar tissue (Fig. 5, 3 independent experiments).

### Genotyping and deletion of the Zip4 gene in the Zip4BKO mice

**Fig 6:**
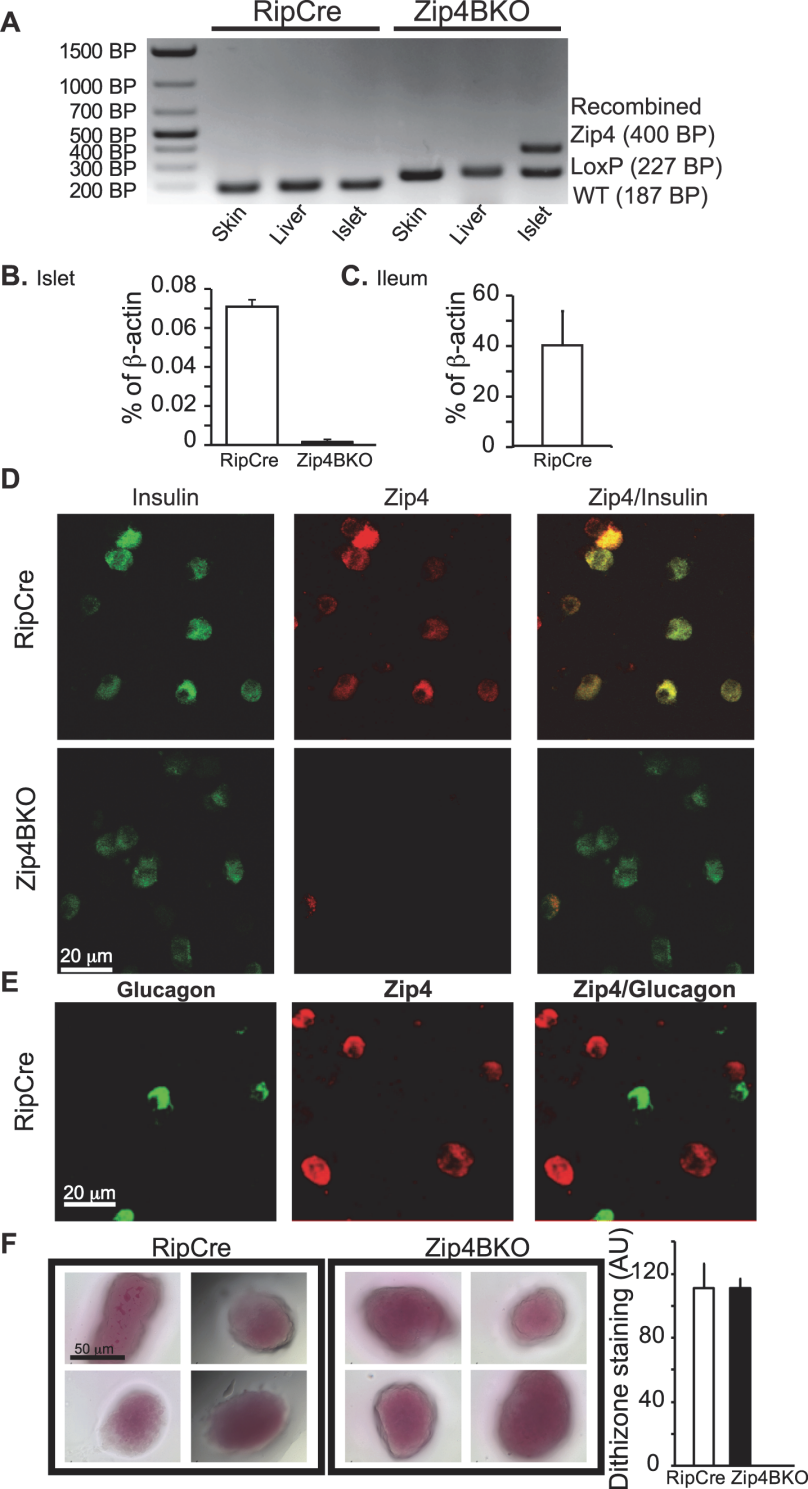
Deletion of Zip4 in Zip4BKO mice. A. PCR results from skin, liver and islet DNA samples showing the wild type (WT), LoxP/LoxP and recombined Zip4 allele. The Zip4 WT allele (187bp) is present in all the different sample of the control RipCre mouse. In the Zip4BKO, LoxP/LoxP Zip4 allele (227bp) is present in all the tissues. Islet DNA of Zip4BKO displays recombined Zip4 gene (400bp) which shows effectiveness and specificity of the Cre lox recombination system. B. qPCR of Zip4 from control RipCre and Zip4BKO isolated islets. C. qPCR was using the ileum as a positive control. D&E immunohistochemistry experiments were performed on dispersed islet cells to co-stain for insulin and Zip4 (D) and glucagon and Zip4 (E). F. Dithizone staining and its quantification in RipCre and Zip4BKO islets.

We generated a beta cell specific Zip4 knockout (Zip4BKO) mouse model as described in the materials and methods (Fig. 1). To confirm the genotype of Zip4BKO mice, genomic DNA was extracted from skin, liver and pancreatic islets and PCR was performed. Flox primers (forward, reverse and post-cre) were used to validate recombination. The wild type, floxed and recombined Zip4 gene corresponded respectively to fragments of 187, 227 and 400 bp in size (Fig. 6A). In RipCre mouse tissues, the only fragment band was the wild type Zip4 fragment. In Zip4BKO skin and liver genomic DNA presented a floxed Zip4 fragment. The genomic DNA from pancreatic islets presented the knock-out fragment which showed the presence of the recombined allele. Moreover, pancreatic islets also displayed a Zip4 floxed fragment originating from non-beta cells (alpha, delta, PP cells, Fig. 6A). qPCR was performed on RipCre and Zip4BKO islets. Zip4 mRNA was detected at low levels in RipCre islets. Nevertheless, Zip4 mRNA was not detected in Zip4BKO islets (Fig. 6B, 3 groups of mice per genotype). Zip4 expression in the ileum was used as a positive control (Fig. 6C, 3 mice). Dispersed islet cells from RipCre and Zip4BKO mice were stained for insulin, glucagon and Zip4. In RipCre mice, insulin and Zip4 were co-localized in numerous cells. In Zip4BKO mice, co-localization was not seen (Fig. 6D). Glucagon positive islet cells were not positive for Zip4 (Fig. 6E). Dithizone staining was performed on isolated mouse islets to assess the islet zinc content. No difference of dithizone staining was observed between RipCre and Zip4BKO islets indicating similar total zinc content (Fig. 6F).

### Glucose homeostasis in Zip4BKO mice

**Fig 7:**
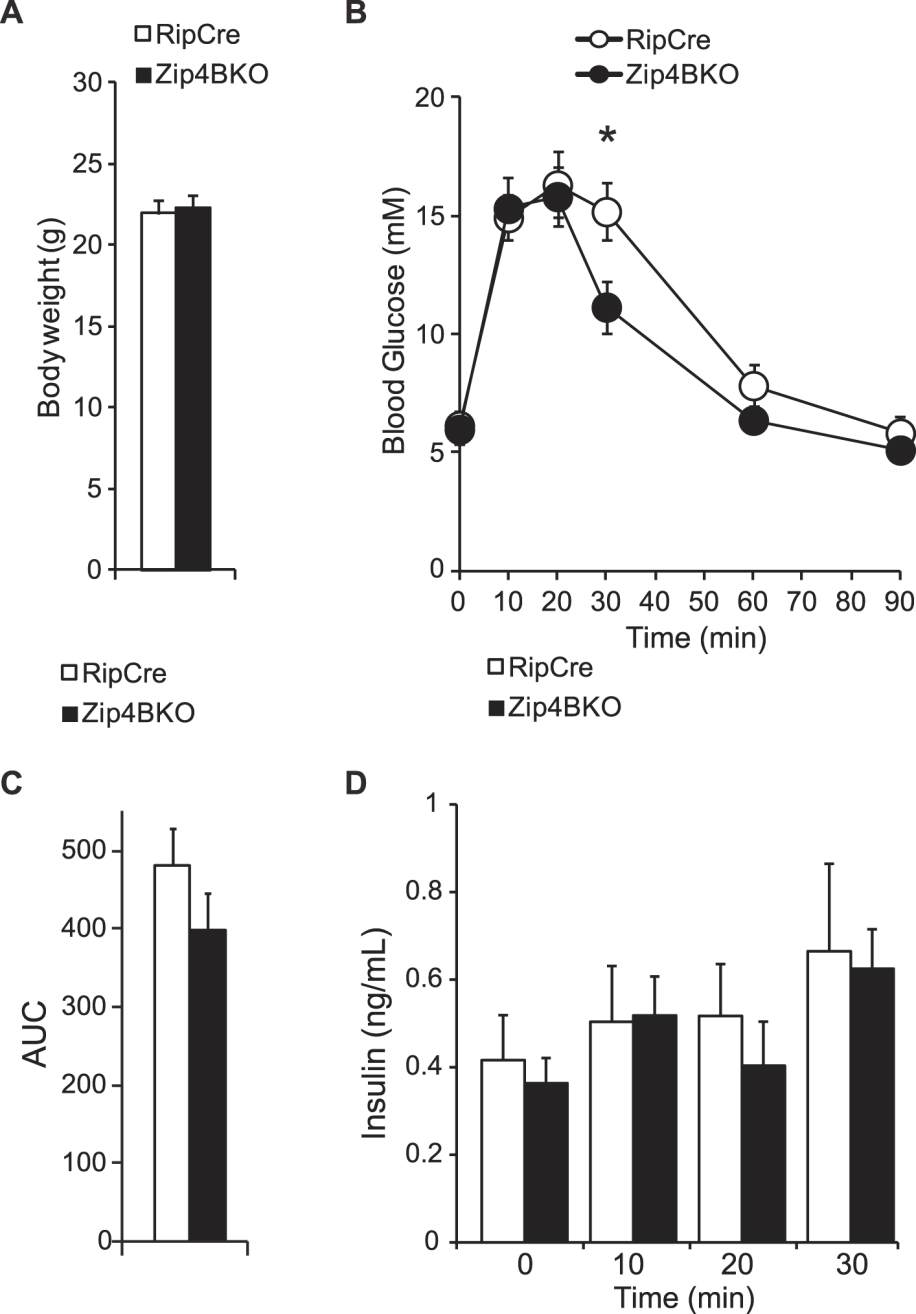
In-vivo characterization of Zip4BKO mice. A. Average body weights of 8 week old RipCre and Zip4BKO mice. B. Oral glucose tolerance test in RipCre and Zip4BKO mice and the corresponding area under the blood glucose curve (C). D. Plasma insulin secretion measured during oral glucose tolerance test. *. p˂0.05.

At 8 weeks of age, RipCre and Zip4BKO mice had similar body weights (Fig. 7A; 7 mice per group). To determine the *in vivo* effect of beta cell specific deletion of Zip4 we performed oral glucose tolerance tests (OGTT) on RipCre and Zip4BKO mice. Zip4BKO mice showed a slight improvement in glucose homeostasis at 30 min (Fig. 7B, 7 mice per group). Nevertheless, there was no difference in the area under the glucose curve (Fig. 7C; 7 mice per group). The corresponding insulin secretion in RipCre and Zip4BKO mice was not changed during the OGTT (Fig. 7D; 5 mice per group).

### Zinc transporter expression in Zip4BKO mouse islets

**Fig 8:**
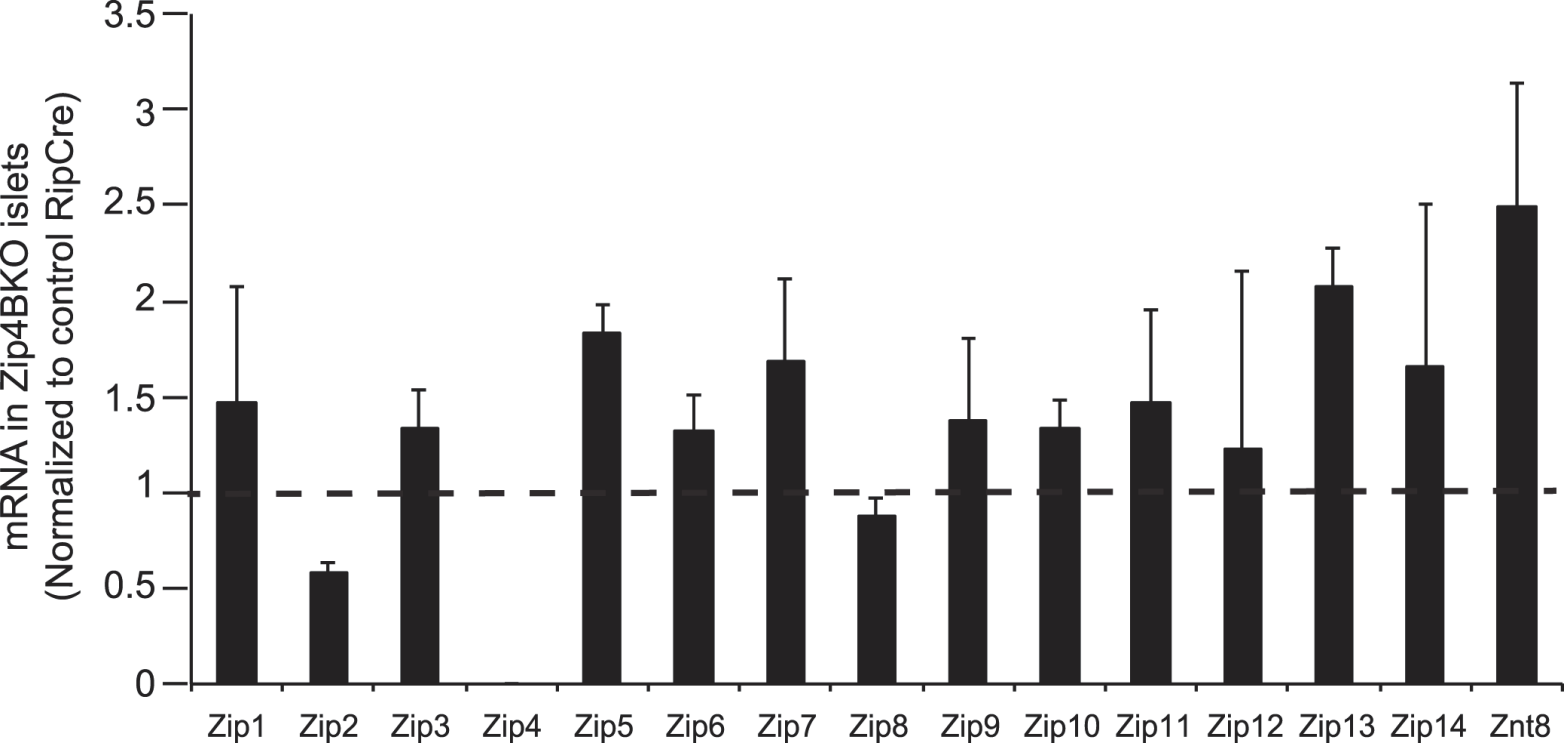
mRNA expression of zinc transporters in islets of Zip4BKO mice. Zip family and Znt8 transcript expression in Zip4BKO mouse islets normalized to control RipCre mouse islets.

Zip1–14 and Znt8 expression was measured in islets from Zip4BKO mice. Expression of most of the Zip transporters was unchanged in Zip4BKO islets. Znt8 mRNA expression was elevated above control, though not significantly (Fig. 8; 3 independent experiments).

## Discussion

The critical role of zinc in insulin biosynthesis in beta cells has been known for more than 30 years [1, 2]. Briefly, zinc assembles with proinsulin to form hexamers inside the golgi apparatus. Following this, proinsulin is directed in early secretory vesicles where it goes through a series of enzymatic cleavages performed by Pc1, Pc2, and CpE enzymes [2, 27, 28]. These enzymatic cleavages result in the formation of c-peptide and insulin. Finally, zinc forms a crystal with insulin allowing the formation of dense cores characterizing fully mature insulin vesicles [1]. Therefore, how zinc enters beta cells represents a critical question in our overall knowledge of zinc in beta cell physiology. To date, little is known about zinc entry in these cells. Zip4 has been localized in human and mouse islet beta cells [18, 19]. However, its function in beta cell physiology is unknown. We hypothesized that Zip4 transports extracellular zinc into beta cells. To study Zip4’s function in zinc transport and insulin secretion in beta cells, we first used mouse insulinoma MIN6 cells. Zip4 overexpression approach was chosen for the following two reasons. First, it has been shown that zinc transporter gene deletion can be associated with compensation mechanisms at the level of gene expression of other zinc transporters [26]. These mechanisms could have masked a specific role of Zip4. Secondly, previous Zip expression profiling performed with qPCR in MIN6 cells revealed that Zip4 is minimally expressed [11, 13, 17, 18]. Zip4 was over-expressed by transfecting MIN6 cells with a pCMV6-Zip4GFP plasmid. Based on a previous Zip4 study in enterocytes, which located Zip4 on their apical surface [19, 29], we anticipated that Zip4 would be located on the plasma membrane of MIN6 cells. Interestingly, our co-localization experiments revealed that overexpressed Zip4 displays a diffuse staining pattern and a lack of specific localization in plasma membrane, endoplasmic reticulum, or mitochondria. Our study was designed to decipher the role of Zip4 under the condition of physiological levels of zinc. Nevertheless, multiple studies showed that zinc deficiency in the diet can increase the expression of Zip4 in enterocytes as well as concentrates its expression in the apical membrane [19, 29–34]. Therefore, we cannot rule out that Zip4 expression and its cellular localization in pancreatic beta cells follows a similar regulation under low zinc conditions.

The Zip transporter group moves zinc into the cytosol from the extracellular compartment and intracellular organelles [14, 15]. Therefore, we expected to obtain higher levels of cytosolic zinc due to the enhanced zinc efflux activity of Zip4. FluoZin-3AM is a cell-permeant zinc fluorescent dye that allowed us to measure accumulation of zinc in the cell cytosol [35]. Fluozin3-AM showed that Zip4 up-regulation increased zinc accumulation in the cytosol. Elevated levels of zinc in the cytosol supports the hypothesis of localization of Zip4 in the plasma membrane. Thus, we cannot completely rule out the hypothesis that some Zip4 was located in the plasma membrane in the Zip4 overexpression condition. It is known that increased granular zinc content can be beneficial for insulin secretion as shown by overexpressing Znt8 in MIN6, which stimulates GSIS [9]. This allows more insulin to be stored in insulin granules and therefore increase insulin secretion under glucose stimulation. In the current study, we used the low affinity zinc dye zinquin, which specifically reflects zinc accumulation in granules [36]. Zinquin fluorescence was significantly higher when Zip4 was upregulated and shows increased accumulation of zinc in MIN6 insulin granules. This may have originated from increased activity of granular Znt8 in order to counteract the elevated cytoplasmic zinc concentration. Therefore, the increased cytosolic zinc accumulation resulted in increased zinc content in insulin granules, thus stimulating insulin secretion.

Zinc is also a regulator of the TCA cycle, mitochondrial transport chain, and glycolytic enzymes [37]. We hypothesized that the altered zinc uptake in mitochondria may modulate the overall mitochondrial respiration in MIN6 cells to promote the insulin secretion increase. To test this, we recorded MMP and did not see any difference in the glucose-induced hyperpolarization of the MMP. Therefore, it is unlikely that the increased glucose stimulated insulin secretion is triggered by a modulation of mitochondrial function.

Insulin biosynthesis and processing were evaluated with the measurements of mRNA expression of Ins1, Pc1, Pc2, CpE and total insulin content in MIN6 cells. qPCR did not reveal increased expression of these genes, nor increased total insulin content. Therefore, it is unlikely that the increased insulin secretion originated from an increased biosynthesis and processing of insulin in MIN6 cells. Moreover, decreased level of Pc2 expression suggested that improper insulin processing might have occurred when Zip4 was upregulated. The assay to quantify insulin measures both pro-and mature insulin. Therefore, defects of insulin processing cannot be ruled out.

Modulation in cytoplasmic zinc levels and paracrine effect of zinc release can be associated with changes in cell death in beta cells [38, 39]. The absence of a significant change in insulin expression and total insulin content suggests that Zip4 is not involved in the zinc-mediated cell death.

Since Zip4 up-regulation *in vitro* in MIN6 cells augmented cytosolic zinc accumulation and insulin secretion and biosynthesis, we wanted to know the role of Zip4 in beta cells *in vivo*. To date, in-situ hybridization [19] and qPCR experiments [18] showed expression of Zip4 mRNA in pancreatic islets. Thus, we wanted to confirm the protein expression of Zip4 in islets. We stained mouse pancreatic sections, which confirmed the presence of Zip4 in islet cells.

We then generated a beta cell-specific Zip4 mouse model. Based on our MIN6 cell data, we expected that loss of Zip4 would reduce cytosolic zinc accumulation and insulin secretion, and thus produce a glucose intolerant phenotype in Zip4BKO mice. Surprisingly, deletion of the Zip4 gene from beta cells in our mouse model resulted in a small improvement of glucose tolerance at 30 min during OGTTs and was not associated with any change in insulin secretion. Overall, our Zip4BKO mouse phenotype did not show Zip4 as mediator of extracellular zinc transport in beta cells. Two hypotheses may explain the absence of this phenotype in the Zip4BKO mouse model. Firstly, Zip4 expression in enterocytes has been shown to be closely linked to zinc content in food [19, 29–34] More precisely, zinc deficiency increases Zip4 abundance in enterocytes and promotes translocation of Zip4 in its apical membrane to increase intestinal zinc absorption. In our study, mice were not deprived of zinc, which might have minimized the impact of the loss of Zip4 on the beta cell physiology. Secondly, compensatory mechanisms originating from increased expression of other zinc transporters have been observed when Znt8 was deleted [26]. In this study, Znt8 expression level was increased when Zip4 was knocked-out which may have allowed beta cells to pump more zinc into insulin granules. This efficient mechanism may have prevented a decrease of granular zinc content and therefore protected insulin secretion. This supports the unchanged total islet zinc content in Zip4BKO observed with dithizone staining.

In conclusion, we first show here that Zip4 protein is located in pancreatic beta cells. Second, Zip4 up-regulation *in-vitro* can increase zinc accumulation in the cytosol and granules of beta cells, leading to stimulated insulin secretion. Third, Zip4 is not essential in mouse for normal beta cell physiology and insulin secretion *in-vivo*.

## References

[1] Emdin SO, Dodson GG, Cutfield JM, Cutfield SM. Role of zinc in insulin biosynthesis. Some possible zinc-insulin interactions in the pancreatic B-cell. Diabetologia. 1980;19(3):174–82. 6997118 10.1007/BF00275265

[2] Dodson G, Steiner D. The role of assembly in insulin's biosynthesis. Current opinion in structural biology. 1998;8(2):189–94. 9631292 10.1016/s0959-440x(98)80037-7

[3] Diabetes Genetics Initiative of Broad Institute of Health, Mit LU, Novartis Institutes of BioMedical Reseach, Saxena R, Voight BF, Lyssenko V, et al Genome-wide association analysis identifies loci for type 2 diabetes and triglyceride levels. Science. 2007;316(5829):1331–6. 17463246 10.1126/science.1142358

[4] Scott LJ, Mohlke KL, Bonnycastle LL, Willer CJ, Li Y, Duren WL, et al A genome-wide association study of type 2 diabetes in Finns detects multiple susceptibility variants. Science. 2007;316(5829):1341–5. 17463248 10.1126/science.1142382 PMC3214617

[5] Sladek R, Rocheleau G, Rung J, Dina C, Shen L, Serre D, et al A genome-wide association study identifies novel risk loci for type 2 diabetes. Nature. 2007;445(7130):881–5. 17293876 10.1038/nature05616

[6] Steinthorsdottir V, Thorleifsson G, Reynisdottir I, Benediktsson R, Jonsdottir T, Walters GB, et al A variant in CDKAL1 influences insulin response and risk of type 2 diabetes. Nature genetics. 2007;39(6):770–5. 17460697 10.1038/ng2043

[7] Zeggini E, Weedon MN, Lindgren CM, Frayling TM, Elliott KS, Lango H, et al Replication of genome-wide association signals in UK samples reveals risk loci for type 2 diabetes. Science. 2007;316(5829):1336–41. 17463249 10.1126/science.1142364 PMC3772310

[8] Chimienti F, Devergnas S, Favier A, Seve M. Identification and cloning of a beta-cell-specific zinc transporter, ZnT-8, localized into insulin secretory granules. Diabetes. 2004;53(9):2330–7. 15331542 10.2337/diabetes.53.9.2330

[9] Chimienti F, Devergnas S, Pattou F, Schuit F, Garcia-Cuenca R, Vandewalle B, et al In vivo expression and functional characterization of the zinc transporter ZnT8 in glucose-induced insulin secretion. Journal of cell science. 2006;119(Pt 20):4199–206. 16984975 10.1242/jcs.03164

[10] Wijesekara N, Dai FF, Hardy AB, Giglou PR, Bhattacharjee A, Koshkin V, et al Beta cell-specific Znt8 deletion in mice causes marked defects in insulin processing, crystallisation and secretion. Diabetologia. 2010;53(8):1656–68. 10.1007/s00125-010-1733-9 20424817 PMC6101216

[11] Nicolson TJ, Bellomo EA, Wijesekara N, Loder MK, Baldwin JM, Gyulkhandanyan AV, et al Insulin storage and glucose homeostasis in mice null for the granule zinc transporter ZnT8 and studies of the type 2 diabetes-associated variants. Diabetes. 2009;58(9):2070–83. 10.2337/db09-0551 19542200 PMC2731533

[12] Lemaire K, Ravier MA, Schraenen A, Creemers JW, Van de Plas R, Granvik M, et al Insulin crystallization depends on zinc transporter ZnT8 expression, but is not required for normal glucose homeostasis in mice. Proceedings of the National Academy of Sciences of the United States of America. 2009;106(35):14872–7. 10.1073/pnas.0906587106 19706465 PMC2736467

[13] Gyulkhandanyan AV, Lee SC, Bikopoulos G, Dai F, Wheeler MB. The Zn2+-transporting pathways in pancreatic beta-cells: a role for the L-type voltage-gated Ca2+ channel. The Journal of biological chemistry. 2006;281(14):9361–72. 16407176 10.1074/jbc.M508542200

[14] Jeong J, Eide DJ. The SLC39 family of zinc transporters. Molecular aspects of medicine. 2013;34(2–3):612–9. 23506894 10.1016/j.mam.2012.05.011 PMC3602797

[15] Eide DJ. Zinc transporters and the cellular trafficking of zinc. Biochimica et biophysica acta. 2006;1763(7):711–22. 16675045 10.1016/j.bbamcr.2006.03.005

[16] Bellomo EA, Meur G, Rutter GA. Glucose regulates free cytosolic Zn(2)(+) concentration, Slc39 (ZiP), and metallothionein gene expression in primary pancreatic islet beta-cells. The Journal of biological chemistry. 2011;286(29):25778–89. 10.1074/jbc.M111.246082 21613223 PMC3138249

[17] Gyulkhandanyan AV, Lu H, Lee SC, Bhattacharjee A, Wijesekara N, Fox JE, et al Investigation of transport mechanisms and regulation of intracellular Zn2+ in pancreatic alpha-cells. The Journal of biological chemistry. 2008;283(15):10184–97. 10.1074/jbc.M707005200 18250168

[18] Wijesekara N, Chimienti F, Wheeler MB. Zinc, a regulator of islet function and glucose homeostasis. Diabetes, obesity & metabolism. 2009;11 Suppl 4:202–14. 10.1111/j.1463-1326.2009.01110.x 19817803

[19] Dufner-Beattie J, Kuo YM, Gitschier J, Andrews GK. The adaptive response to dietary zinc in mice involves the differential cellular localization and zinc regulation of the zinc transporters ZIP4 and ZIP5. The Journal of biological chemistry. 2004;279(47):49082–90. 15358787 10.1074/jbc.M409962200

[20] Geiser J, Venken KJ, De Lisle RC, Andrews GK. A mouse model of acrodermatitis enteropathica: loss of intestine zinc transporter ZIP4 (Slc39a4) disrupts the stem cell niche and intestine integrity. PLoS genetics. 2012;8(6):e1002766 10.1371/journal.pgen.1002766 22737083 PMC3380849

[21] Lee SC, Robson-Doucette CA, Wheeler MB. Uncoupling protein 2 regulates reactive oxygen species formation in islets and influences susceptibility to diabetogenic action of streptozotocin. The Journal of endocrinology. 2009;203(1):33–43. 10.1677/JOE-09-0117 19635759

[22] Hardy AB, Fox JE, Giglou PR, Wijesekara N, Bhattacharjee A, Sultan S, et al Characterization of Erg K+ channels in alpha- and beta-cells of mouse and human islets. The Journal of biological chemistry. 2009;284(44):30441–52. 10.1074/jbc.M109.040659 19690348 PMC2781599

[23] Miyazaki J, Araki K, Yamato E, Ikegami H, Asano T, Shibasaki Y, et al Establishment of a pancreatic beta cell line that retains glucose-inducible insulin secretion: special reference to expression of glucose transporter isoforms. Endocrinology. 1990;127(1):126–32. 2163307 10.1210/endo-127-1-126

[24] Huang ZL, Dufner-Beattie J, Andrews GK. Expression and regulation of SLC39A family zinc transporters in the developing mouse intestine. Developmental biology. 2006;295(2):571–9. 16682017 10.1016/j.ydbio.2006.03.045

[25] Schneider CA, Rasband WS, Eliceiri KW. NIH Image to ImageJ: 25 years of image analysis. Nature methods. 2012;9(7):671–5. 22930834 10.1038/nmeth.2089 PMC5554542

[26] Hardy AB, Wijesekara N, Genkin I, Prentice KJ, Bhattacharjee A, Kong D, et al Effects of high-fat diet feeding on Znt8-null mice: differences between beta-cell and global knockout of Znt8. American journal of physiology Endocrinology and metabolism. 2012;302(9):E1084–96. 10.1152/ajpendo.00448.2011 22338079 PMC3774340

[27] Steiner DF, Rouille Y, Gong Q, Martin S, Carroll R, Chan SJ. The role of prohormone convertases in insulin biosynthesis: evidence for inherited defects in their action in man and experimental animals. Diabetes & metabolism. 1996;22(2):94–104. 8792089

[28] Goodge KA, Hutton JC. Translational regulation of proinsulin biosynthesis and proinsulin conversion in the pancreatic beta-cell. Seminars in cell & developmental biology. 2000;11(4):235–42. 10966857 10.1006/scdb.2000.0172

[29] Kim BE, Wang F, Dufner-Beattie J, Andrews GK, Eide DJ, Petris MJ. Zn2+-stimulated endocytosis of the mZIP4 zinc transporter regulates its location at the plasma membrane. The Journal of biological chemistry. 2004;279(6):4523–30. 14612438 10.1074/jbc.M310799200

[30] Dufner-Beattie J, Wang F, Kuo YM, Gitschier J, Eide D, Andrews GK. The acrodermatitis enteropathica gene ZIP4 encodes a tissue-specific, zinc-regulated zinc transporter in mice. The Journal of biological chemistry. 2003;278(35):33474–81. 12801924 10.1074/jbc.M305000200

[31] Wang K, Zhou B, Kuo YM, Zemansky J, Gitschier J. A novel member of a zinc transporter family is defective in acrodermatitis enteropathica. American journal of human genetics. 2002;71(1):66–73. 12032886 10.1086/341125 PMC419995

[32] Weaver BP, Dufner-Beattie J, Kambe T, Andrews GK. Novel zinc-responsive post-transcriptional mechanisms reciprocally regulate expression of the mouse Slc39a4 and Slc39a5 zinc transporters (Zip4 and Zip5). Biological chemistry. 2007;388(12):1301–12. 18020946 10.1515/BC.2007.149 PMC2376820

[33] Kambe T, Andrews GK. Novel proteolytic processing of the ectodomain of the zinc transporter ZIP4 (SLC39A4) during zinc deficiency is inhibited by acrodermatitis enteropathica mutations. Molecular and cellular biology. 2009;29(1):129–39. 10.1128/MCB.00963-08 18936158 PMC2612479

[34] Liuzzi JP, Bobo JA, Lichten LA, Samuelson DA, Cousins RJ. Responsive transporter genes within the murine intestinal-pancreatic axis form a basis of zinc homeostasis. Proceedings of the National Academy of Sciences of the United States of America. 2004;101(40):14355–60. 15381762 10.1073/pnas.0406216101 PMC521973

[35] Gee KR, Zhou ZL, Ton-That D, Sensi SL, Weiss JH. Measuring zinc in living cells. A new generation of sensitive and selective fluorescent probes. Cell calcium. 2002;31(5):245–51. 12098227 10.1016/S0143-4160(02)00053-2

[36] Qian WJ, Aspinwall CA, Battiste MA, Kennedy RT. Detection of secretion from single pancreatic beta-cells using extracellular fluorogenic reactions and confocal fluorescence microscopy. Analytical chemistry. 2000;72(4):711–7. 10701254 10.1021/ac991085t

[37] Dineley KE, Votyakova TV, Reynolds IJ. Zinc inhibition of cellular energy production: implications for mitochondria and neurodegeneration. Journal of neurochemistry. 2003;85(3):563–70. 12694382 10.1046/j.1471-4159.2003.01678.x

[38] Kim BJ, Kim YH, Kim S, Kim JW, Koh JY, Oh SH, et al Zinc as a paracrine effector in pancreatic islet cell death. Diabetes. 2000;49(3):367–72. 10868957 10.2337/diabetes.49.3.367

[39] Formigari A, Irato P, Santon A. Zinc, antioxidant systems and metallothionein in metal mediated-apoptosis: biochemical and cytochemical aspects. Comparative biochemistry and physiology Toxicology & pharmacology: CBP. 2007;146(4):443–59. 17716951 10.1016/j.cbpc.2007.07.010

